# Structural insights into the modulatory role of the accessory protein WYL1 in the Type VI-D CRISPR-Cas system

**DOI:** 10.1093/nar/gkz269

**Published:** 2019-04-12

**Authors:** Heng Zhang, Cheng Dong, Li Li, Gregory A Wasney, Jinrong Min

**Affiliations:** 1 Structural Genomics Consortium, University of Toronto, Toronto, ON M5G 1L7, Canada; 2 The Hospital for Sick Children, Toronto, ON M5G 0A4, Canada; 3 Department of Physiology, University of Toronto, Toronto, ON M5S 1A8, Canada

## Abstract

The Type VI-D CRISPR-Cas system employs an RNA-guided RNase Cas13d with minimal targeting constraints to combat viral infections. This CRISPR system contains RspWYL1 as a unique accessory protein that plays a key role in boosting its effector function on target RNAs, but the mechanism behind this RspWYL1-mediated stimulation remains completely unexplored. Through structural and biophysical approaches, we reveal that the full-length RspWYL1 possesses a novel three-domain architecture and preferentially binds ssRNA with high affinity. Specifically, the N-terminus of RspWYL1 harbors a ribbon-helix-helix motif reminiscent of transcriptional regulators; the central WYL domain of RspWYL1 displays a Sm-like β-barrel fold; and the C-terminal domain of RspWYL1 primarily contributes to the dimerization of RspWYL1 and may regulate the RspWYL1 function via a large conformational change. Collectively, this study provides a first glimpse into the complex mechanism behind the RspWYL1-dictated boosting of target ssRNA cleavage in the Type VI-D CRISPR-Cas system.

## INTRODUCTION

The adaptive immune responses of bacteria and archaea utilize CRISPR-Cas (clustered regularly interspaced short palindromic repeats and CRISPR-associated proteins) systems as their adaptive immune responses to combat viruses and harmful mobile genetic elements through RNA-mediated, sequence-specific targeting mechanisms (1). Upon activation of the CRISPR-Cas defense systems, the host genome incorporates short segments of foreign invading DNA as variable spacer sequences between invariable pseudo-palindromic repeat sequences (2,3). Transcription of the resulting CRISPR array leads to the formation of long precursor CRISPR RNA (pre-crRNA). These pre-crRNA can be subsequently processed into small mature crRNA that can serve as guides for the CRISPR-Cas immunity to precisely target and degrade invading foreign nucleic acids based on sequence complementarity (4,5).

Due to their considerable diversity, the CRISPR-Cas systems have been classified into two classes and six types with many subtypes (6,7). In contrast to the multi-subunit effector complexes in Class 1 systems, the more compact Class 2 systems employ a large single multi-domain effector coupled with crRNA to target foreign DNA or RNA for interference (8,9). As the latest identified member of the Class 2 CRISPR systems, the Type VI-D CRISPR-Cas system employs the effector Cas13d (the smallest CRISPR effector among all known Type VI systems) that contains two HEPN (higher eukaryotes and prokaryotes nucleotide-binding) domains (10,11). Owing to the RNase activity of the HEPN domains, the Type VI-D CRISPR-Cas immune response specifically targets single-stranded foreign RNA for destruction (cis-cleavage) (9,12,13). Interestingly, the target RNA recognition by Cas13d would stimulate the non-specific collateral RNase activity of Cas13d (trans-cleavage) (9,12,14).

In addition to its compact size relative to the other known CRISPR systems, the most unique feature of the Type VI-D CRISPR-Cas system is highlighted by its associated regulatory module involving the accessory protein WYL1. The *Ruminococcus sp*. WYL1 (RspWYL1) has been demonstrated to significantly boost Cas13d’s target selectivity and RNase activity (both the cis- and trans-cleavage) (9). RspWYL1 is capable of augmenting Cas13d’s ssRNA cleavage activity in a RspWYL1 protein-dose dependent manner *in vitro* (9), implying a dynamic nature of this positive modulatory mechanism on the Type VI-D CRISPR-Cas system. RspWYL1 and Csx28 are to date the only known molecules that can positively modulate CRISPR-Cas adaptive immune responses (9,15). Notably, this RspWYL1-mediated boosting is a universal mechanism for divergent Cas13d orthologs, highlighting the significance of this molecule in the evolution of prokaryotic adaptive immune systems as well as in the CRISPR-Cas genome editing toolkit. To further add a layer of complexity to the CRISPR-Cas systems, a WYL domain-containing protein can negatively regulate the Type I-D CRISPR-Cas system through transcriptional control of the CRISPR expression (16). Additionally, a recent study suggests that the WYL domain of *Thermotoga elfii* PIF1 helicase preferentially binds ssDNA. More importantly, mutations of the predicted ssDNA-binding site in PIF1 diminish its DNA helicase activity while increasing ATPase activity (17), implying a central functional theme of the WYL domain in binding nucleic acid and regulating enzymatic activity. Collectively, the WYL domain likely represents a general modulatory component of various CRISPR-Cas systems with either a stimulatory or inhibitory effect on the respective effector enzyme in a context-dependent manner.

Modulation of the regulatory elements of known CRISPR-Cas systems constitutes an efficient means to expand the versatility of CRISPR-Cas applications for genomic engineering. The pivotal roles of WYL domain-containing proteins in modulating CRISPR-Cas systems underline an urgent need to delineate their respective molecular mechanisms so that we can re-engineer these modules with accuracy and precision. Here, we present the crystal structure of the full-length RspWYL1 protein, and biophysical evidences demonstrating that RspWYL1 functions as a homodimer in solution and harbors single-stranded RNA (ssRNA) binding activity with high affinity. Moreover, RspWYL1 directly interacts with Cas13d effector. We propose that RspWYL1 might serve as an ssRNA-specific sequence-independent anchor to capture potential RNA substrates and then present these target ssRNA to be examined by the CRISPR-Cas13d system, thereby leading to a RspWYL1-dose dependent increase of the efficiency of identifying foreign invading RNAs during a Type VI-D CRISPR-Cas-mediated adaptive immune response. The structural and biophysical data of RspWYL1 presented in this study will provide valuable information in the designs of modified Type VI-D CRISPR-Cas systems to suit various genome editing applications.

## MATERIALS AND METHODS

### Protein expression and purification

The DNA sequence encoding full-length RspWYL1 was cloned into pET28-MKH8SUMO vector using the InFusion^™^ cloning kit (ClonTech) according to the manufacturer’s instructions. The plasmid contains an N-terminal His_8_-SUMO followed by a TEV cleavage site. The RspCas13d and EsCas13d constructs, pET28a-MH6-RspCas13d and pET28a-MH6-EsCas13d respectively, were obtained from Addgene (9). Recombinant proteins of RspWYL1, RspWYL1 mutants, RspCas13d or EsCas13d were expressed in *Escherichia coli* BL21(DE3) cells in Terrific Broth medium and induced with 0.5 mM Isopropyl β-D-1-thiogalactopyranoside (IPTG) at 16°C overnight. Cells were lysed by sonication in 20 mM Tris–HCl, pH 7.5, 500 mM NaCl, 5% (v/v) glycerol and 5 mM β-mercaptoethanol. The crude extracts were cleared by centrifugation at 15 000 rpm for 1 h. The supernatants were loaded on Ni-NTA resins and washed with 20 mM Tris–HCl, pH 7.5, 500 mM NaCl, 5% (v/v) glycerol, 25 mM imidazole and 5 mM β-mercaptoethanol, and the respective proteins were eluted using elution buffer containing 20 mM Tris–HCl, pH 7.5, 500 mM NaCl, 5% (v/v) glycerol, 250 mM imidazole and 5 mM β-mercaptoethanol. The SUMO or MBP tags were cleaved by TEV proteases in the dialysis buffer (20 mM Tris–HCl, pH 7.5, 200 mM NaCl and 5 mM β-mercaptoethanol) at 4°C overnight, and then the samples were re-loaded on Ni-NTA resins to remove the tags and TEV proteases. Subsequently, the RspWYL1, RspWYL1 mutants, RspCas13d or EsCas13d proteins were further purified by Heparin chromatography (HiTrap Heparin HP, GE Healthcare), followed by size-exclusion chromatography (Superdex 200 10/300, GE Healthcare) in 20 mM Tris-HCl, pH 7.5, 150 mM NaCl and 1 mM 1,4-dithiothreitol (DTT). Protein purity was analyzed by sodium dodecyl sulphate-polyacrylamide gel electrophoresis (SDS-PAGE) with Coomassie blue staining. The final purified RspWYL1, RspWYL1 mutants, RspCas13d or EsCas13d proteins were concentrated to 10–20 mg/ml, flash-frozen in liquid nitrogen and then stored at −80°C. The SeMet-labeled RspWYL1 proteins were obtained by overexpression using the prepacked M9 SeMet growth media kit (Medicilon) following manufacturer’s instructions, and were purified by the same protocol as above.

### Crystallization and structure determination

SeMet-labeled and native RspWYL1 crystals were grown at 18°C using sitting-drop vapor diffusion method by mixing 0.5 μl protein and 0.5 μl reservoir solution (0.1 M ammonium acetate, 0.015 M magnesium acetate tetrahydrate, 0.05 M sodium cacodylate trihydrate, pH 6.5, 10% v/v 2-propanol). The crystal was cryoprotected in reservoir solution supplemented with 20% (v/v) glycerol and flash frozen in liquid nitrogen for data collection.

Diffraction data were collected at beam line 19-ID of the Advanced Photon Source, reduced with XDS (18) and merged with AIMLESS (19). The SeMet-labeled and native RspWYL1 structures were solved by single wavelength anomalous diffraction with XPREP and SHELX software and molecular replacement (20,21). Automatic model building was begun with BUCCANEER (22) and continued with ARP/wARP (23). Manual model re-building, restrained refinement and geometry analysis were performed with COOT (24), REFMAC (25) and PHENIX.MOLPROBITY (26), respectively.

### Isothermal titration calorimetry (ITC)

Isothermal titration calorimetry (ITC) measurements were performed with the ITC200 microcalorimeter (MicroCal) at 25°C. The purified RspWYL1, RspWYL1 mutants, RspCas13d and EsCas13d proteins were extensively dialyzed against the ITC buffer (50 mM Tris–HCl, pH 7.5, 100 mM NaCl and 0.5 mM TCEP) and then diluted to 0.05–0.1 mM using the ITC buffer, followed by loading into the cell chamber of the microcalorimeter. Synthesized RNAs/DNAs (IDT) and purified RspWYL1 proteins were diluted to 0.5–1 mM using the ITC buffer for loading into the springe, and then 19 injections of RNAs, DNAs or RspWYL1 proteins were carried out with a spacing of 180 s and a reference power of 15 ucal/s. At least two independent experiments were performed. OriginLab Pro7 software was employed for data analysis.

### HPLC size-exclusion chromatography coupled with multi-angle light scattering (HPLC SEC-MALS)

HPLC SEC-MALS was performed on a Wyatt MiniDAWN TREOS coupled with Wyatt Optilab T-rEX detection system connected to an Agilent 1260 Infinity II Bio-Inert HPLC System using an AdvanceBio SEC 300A metal column (Agilent). The metal column was pre-equilibrated in the HPLC buffer (50 mM Tris-HCl, pH 7.5, 150 mM NaCl and 1 mM TCEP) overnight, and then a bovine serum albumin solution was used for aligning the signals and standardizing peaks. Purified RspWYL1 proteins were extensively dialyzed against the HPLC buffer and then adjusted to 10 mg/ml for loading onto the HPLC SEC-MALS system. Three independent experiments using three independently purified RspWYL1 protein samples were performed. ASTRA software (Wyatt) was employed for data processing.

## RESULTS AND DISCUSSION

### Overall structure of RspWYL1

**Figure 1.**
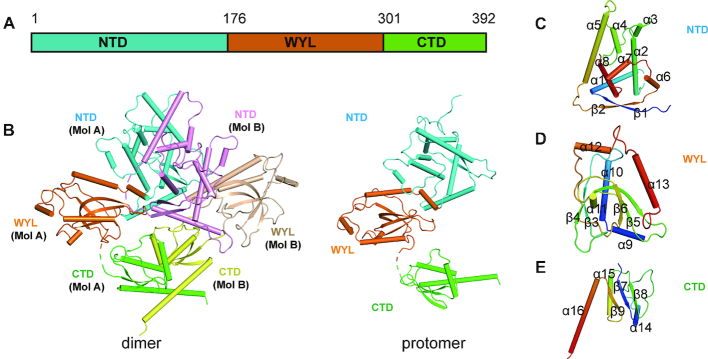
Overall structure of full-length RspWYL1. (**A**) Schematic representation of the full-length RspWYL1 (amino acids 1–392). NTD: N-terminal domain. CTD: C-terminal domain. (**B**) Overall structure of RspWYL1 in ribbon representation. Left panel: Two RsWYL1 molecules (Mol A and Mol B) are present in each asymmetric unit. The NTD and CTD are shown in cyan (or pink) and green (or yellow), respectively. The WYL domain is shown in orange (or wheat). Right panel: Overall structure of a single RspWYL1 molecule. (**C–E**) Subdomains of RspWYL1 are rainbow-colored.

To gain mechanistic insights into the modulatory role of WYL1 in the newly discovered Type VI-D CRISPR-Cas system, we determined the crystal structure of the full-length WYL1 protein from *Ruminococcus sp*. (RspWYL1) by the single-wavelength anomalous dispersion (SAD) method (Figure 1A and Table 1). Each asymmetric unit in the crystal structure contains two RspWYL1 molecules, forming a diamond-shaped, ∼95 × 85 × 80 Å homodimer (Figure 1B, left panel). The RspWYL1 protomer comprises three domains, which are arranged in a triangular fashion (Figure 1B, right panel). The N-terminal domain (NTD) of RspWYL1 spanning residues 1–176 consists of a cluster of α-helices flanked by an antiparallel β-sheet (Figure 1C). The middle WYL domain of RspWYL1, comprising residues 177–301, adopts an antiparallel four-stranded β-barrel fold (Figure 1D). The C-terminal domain (CTD) adopts a two-layer αβ sandwich fold, in which a three-stranded β-sheet packs beneath the helices (Figure 1E). The two protomers in the RspWYL1 dimer spatially arrange side-by-side, and interact with each other mainly through their NTD and CTD domains (Figure 1B).

**Table 1.** X-ray diffraction and refinement statistics

Data collection	
Space group	P2_1_
Unit cell constants *a, b, c, β*	76.8Å, 95.3Å, 77.3Å, 112.8°
Resolution limits (outer shell)	47.65–2.10 (2.15–2.10)
Completeness [%]	99.2 (98.7)
*R*_merge_	0.077 (1.371)
Mean I/sigma	16.0 (1.3)
Multiplicity	6.7 (6.6)
Refinement	
Resolution limits [Å]	39.5 - 2.2
Number of HKLs (work/free)	49263/2554
*R*_work_/*R*_free_	0.212/0.247
Number of atoms all / protein	6123 / 6010
Average *B*-factor all / protein	52.2 / 52.4
RMSD bonds / angles	0.010Å / 1.4°

Values in parentheses are for highest resolution shell.

### RspWYL1 dimerizes mainly through its CTD

**Figure 2.**
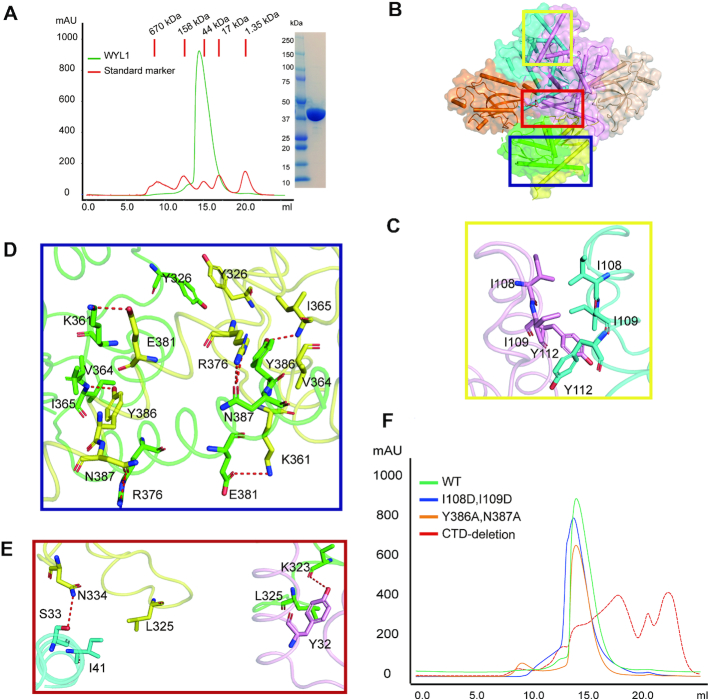
The CTD–CTD intermolecular interactions provide the major driving forces for homodimerization. (**A**) RspWYL1 behaves as a dimer in solution. (Left) Overlaid size-exclusion chromatograms (SEC) of full-length RspWYL1 (amino acids 1–392) and protein molecular mass standard mixture solution. (Right) SDS-PAGE analysis of the SEC elution fractions corresponding to RspWYL1 is shown with the standard protein marker. (**B**) Surface representations of the RspWYL1 homodimer. The interaction surfaces of NTD–NTD, NTD–CTD and CTD–CTD are indicated by yellow, red and blue boxes, respectively. (**C–E**) A close-up view of the intermolecular polar and hydrophobic interactions at the homo-dimeric interface. Hydrogen bonds are represented by red dashed lines. (**F**) Overlaid size-exclusion chromatograms of RspWYL1 WT and mutants.

Protein oligomerization has functional implications in a variety of biological processes (27,28,29). The full-length RspWYL1 protein eluted at a volume equivalent to a molecular weight of ∼90 kDa on a Superdex 200 gel filtration column, matching the theoretical molecular weight of dimeric RspWYL1, suggesting that RspWYL1 exists as a dimer in solution (Figure 2A). Multi-angle light scattering studies further supported the dimer model (Supplementary Figure S1). The two protomers have an almost parallel arrangement with an ∼2190 Å^2^ surface area buried upon dimerization (30).

Residues from both NTD and CTD of RspWYL1 constitute the homodimerization interface. Multiple hydrophobic and polar interactions occur at three major intermolecular interfaces (NTD–NTD, CTD–CTD and NTD–-CTD) (Figure 2B). In particular, the respective hydrophobic residues Ile108, Ile109 and Tyr112 of RspWYL1 lining in the NTD–NTD interface pack closely against each other, forming an intermolecular hydrophobic core (Figure 2C). On the other hand, the CTD−CTD interactions involve 16 residues to generate an interface area of ∼1300 Å^2^, accounting for the majority of intermolecular interactions of the RspWYL1 homodimer (∼60%) (30). Specifically, Lys361 forms salt bridges with Glu381 from the other molecule, while the guanidino group of Arg376 forms hydrogen bonds with the side chain of Asn387 of the other molecule (Figure 2D). Furthermore, the hydroxyl group of Tyr386 is hydrogen bonded to the backbone of Ile365 of the other molecule. These polar interactions are further stabilized by hydrophobic interactions between Tyr386 and Val364 and the π–π stacking interactions between the two Tyr326 residues of the RspWYL1 dimer (Figure 2D).

The symmetric NTD–NTD and CTD–CTD binding interfaces are further enforced by the intermolecular NTD–CTD contacts as well. Tyr32 and Ile41 in the NTD of one protomer engage in hydrophobic interactions with Leu325 in the CTD of the other protomer (Figure 2E). Altogether, the RspWYL1 homodimer formation is mediated by multiple intermolecular hydrophobic and polar interactions. The double mutant Y386A/N387A or I108D/I109D still behaves as a homodimer on gel filtration, confirming the extensive interactions between the two protomers (Figure 2F). The ΔCTD mutant (aa 3–301) is unstable (most proteins precipitate) in solution (Figure 2F and Supplementary Figure S2), indicating the importance of the CTD in stabilizing the RspWYL1 proteins. Our results indicate that CTD–CTD interactions constitute the major intermolecular driving force to maintain RspWYL1 as a homodimer in solution.

### The NTD of RspWYL1 contains atypical ribbon–helix–helix motifs

**Figure 3.**
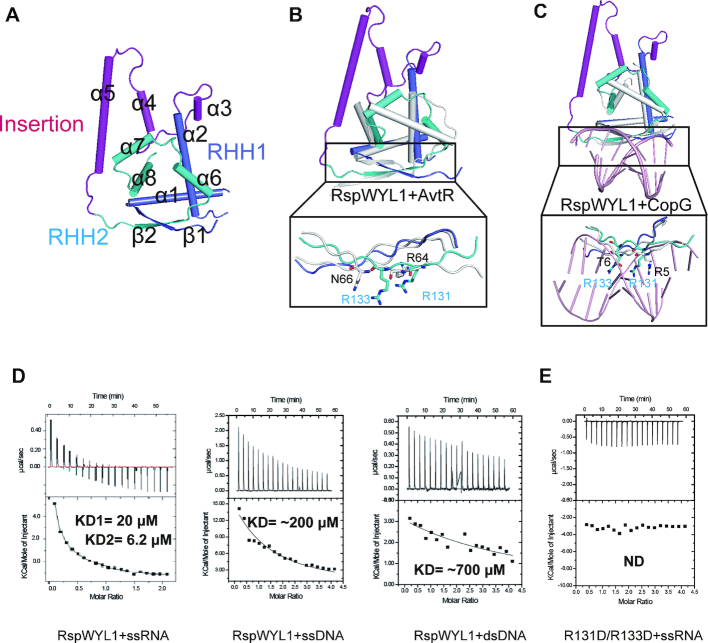
The NTD of RspWYL1 harbors non-canonical tandem RHH motifs with a novel helical insertion. (**A**) NTD contains two RHH motifs. RHH1, RHH2 and the helical insertion are colored blue, cyan and magenta, respectively. (**B**) Superposition of the RHH regions of NTD and AvtR (gray, PDB: 4VH0). The residues involved in the nucleic acid binding are shown in stick representation. (**C**) Superposition of the RHH regions of NTD and CopG (gray)-DNA (pink) complex (PDB: 1B01). The residues involved in the nucleic acid binding are shown in stick representation. (**D**) Isothermal titration calorimetric (ITC) analysis of RspWYL1’s binding capabilities toward various types of nucleic acids. ND: no detectable binding. (**E**) The double mutant on RHH (R131D/R133D) impairs the ssRNA binding.

Ribbon–helix–helix (RHH) motifs are often found in bacterial transcription factors (31). The NTD of RspWYL1 harbors two RHH motifs, denoted RHH1 and RHH2. RHH1 consists of α1, α2 and β1, while RHH2 encompasses α6, α7, α8 and β2 (Figures 1C and 3A). These helices pack against each other and form hydrophobic cores, a common feature of the RHH superfamily (32). A known characteristic of the RHH motif is that it forms a tightly intertwined homodimer with a 2-fold symmetry. However, the two RHH motifs of RspWYL1 are in a single polypeptide chain and fold together in a pseudo-2-fold symmetry, highlighting the diversity of the RHH superfamily. These two RHH motifs align well with each other (Supplementary Figure S3A), resulting in a root mean square deviation (RMSD) of 2.791 Å.

A Dali search identified the transcriptional regulator AvtR from *Acidianus* filamentous virus and the transcriptional repressor CopG from *Streptococcus agalactiae* as the closest structural homologous to the RHH motifs of RspWYL1 (RspWYL1^RHH^) (33). Interestingly, AvtR also contains two RHH motifs in a single polypeptide chain similar to RspWYL1^RHH^ (34). Although RspWYL1^RHH^ has an overall fold similar to those homologs, detailed comparisons revealed several unique features of RspWYL1^RHH^. First, a helical insertion comprising helices α3–5 between RHH1 and RHH2 is present in RspWYL1^RHH^ (Figure 3A–C). The helical insertion is involved in the RspWYL1 dimerization and intramolecular RHH–WYL interactions (Figure 2C and E). Second, in contrast to the canonical RHH topology of β1–α1–α2 found in AvtR and CopG (31,34), two helices (α6 and α7) equivalent to α1 are observed in the RHH2 motif of RspWYL1 (Figure 3A–C). Finally, the most notable differences between RspWYL1^RHH^ and its structural homologs exist in the central β-sheet implicated in binding the major groove of dsDNA (32). In the RHH superfamily, a positively charged residue is always present at the start or end (position 2 or 6, respectively) of the β-stands, and an uncharged polar residue is present at position 4, conferring some sequence-specific nucleotide base contacts (31,32,34). In the RHH2 of RspWYL1, a positively charged Arg131 is present at the position 2 (Figure 3B and C). Instead, an unexpected charged residue (Arg133) is present at the position 4 that would cause steric clashes with the major groove of dsDNA, implying that RspWYL1 might disfavor dsDNA. Indeed, consistent with the modulation role of RspWYL1 in the crRNA-targeted ssRNA cleavage, our ITC results showed that RspWYL1 displays a much higher binding affinity for ssRNA than ssDNA or dsDNA (Figure 3D and Supplementary Table S1). Additionally, the double mutant R131D/R133D of RspWYL1 failed to bind ssRNA (Figure 3E). It is likely that the ssRNA binding event of RspWYL1 may be coupled to the stimulatory effect of RspWYL1 on target RNA cleavage; therefore, the R131D/R133D mutant would be expected to impair the stimulatory effect. Collectively, RspWYL1^RHH^ represents a non-canonical RHH structure unique to the Type VI-D CRISPR-Cas system. Based on our solved RspWYL1 structure, we propose that RspWYL1^RHH^ may function as a scaffold domain or facilitate the base-pairing between crRNA and target RNA (9), and thus future investigations into this non-canonical role of RspWYL1^RHH^ would be worthwhile.

### Structural features of the WYL domain of RspWYL1

Although the biological functions of the WYL domain have remained largely underexplored (17,35,36), the strong evolutionary selection pressure to conserve WYL domain-containing proteins in Type VI-D CRISPR-Cas systems highlights the significance of the WYL domain in the Type VI-D CRISPR-Cas mediated adaptive immunity (9). Our structural analysis revealed that the WYL domain projects out from the dimerization interface of the RspWYL1 homodimer (Figure 1B), and adopts a Sm-like β-barrel structure (Figure 1D). In accordance with its known implication in binding nucleic acids (17,35,36), this structural feature of the WYL domain would easily enable the accommodation of a target ssRNA or crRNA as its interacting ligand (Figure 1B).

**Figure 4.**
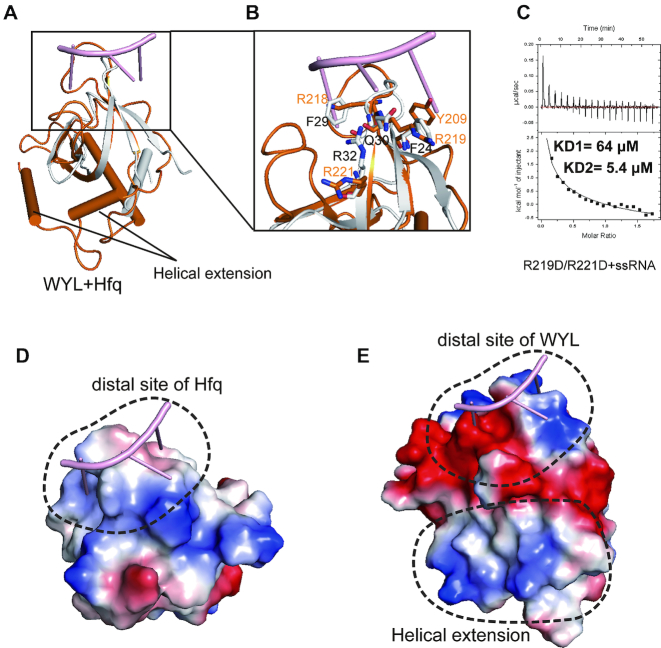
Structural features of the WYL domain of RspWYL1. (**A**) Structural comparison of the WYL domain of RspWYL1 (orange) and BsHfq (gray)-RNA (pink) complex (PDB: 3HSB). (**B**) Four residues Phe24, Phe29, Asn30 and Arg32 in BsHfq are responsible for recognition of RNA. Arg32 in BsHfq plays a significant role in binding RNA. (**C**) ITC binding curves of the R219D/R221D mutant with ssRNA. (**D**) Electrostatic surface representations of BsHfq. (**E**) Electrostatic surface representations of the WYL domain of RspWYL1. The helical extension of WYL domain is positively charged.

Our Dali search indicated that the WYL domain of RspWYL1 has a high structural similarity to the bacterial post-transcriptional regulator Hfq, which is known to bind small RNAs in the mRNA decay pathway (33,37,38) (Figure 4A). Structural comparison of the WYL domain and the *Bacillus subtilis* Hfq–RNA complex provided us with an opportunity to gain insights into how the WYL domain of RspWYL1 recognizes ssRNA species (Figure 4A and B) (38). Whereas two major separate binding sites (proximal and distal sites) in Hfq participate in ssRNA binding (37,38), the region in the WYL domain of RspWYL1 corresponding to the proximal site is blocked due to the homodimerization between the NTD–CTD interface (Supplementary Figure S3B). Given that the distal site of Hfq preferentially binds to purine-rich sequences independently of the proximal site (37,38), the equivalent solvent-exposed region in the WYL domain could easily accommodate ssRNA (Supplementary Figure S3B). The conserved residues Asn30 and Arg32 on the distal site of *Bacillus subtilis* Hfq (BsHfq) play key roles in mediating ssRNA binding (Figure 4B) (38); however, double mutation of the equivalent residues (R219D/R221D) on RspWYL1 did not disrupt the ssRNA binding (Figure 4C), indicating a distinct ssRNA binding mode from Hfp.

In spite of some similarities, noteworthy differences exist between the distal sites of RspWYL1 and BsHfq (Figure 4). First, the aromatic residue Phe29 in BsHfq stacking with the ring of nucleotide base is replaced by the positively charged Arg218 in RspWYL1 (Figure 4B). Second, whereas the distal site of BsHfq displays a positive electrostatic potential (Figure 4D), the equivalent site in the WYL domain of RspWYL1 is highly negatively charged (Figure 4E), implying a unique ssRNA-recognition mode for RspWYL1. Finally, compared with Hfq, two additional α-helices are present in the WYL domain of RspWYL1 (Figure 4A), which form an extensive, positively charged surface (Figure 4E). These distinct structural features imply that the WYL domain of RspWYL1 may exhibit a unique ssRNA-recognition mode distinct from that of Hfq.

### Implications of CTD in the modulation of RspWYL1’s activity

**Figure 5.**
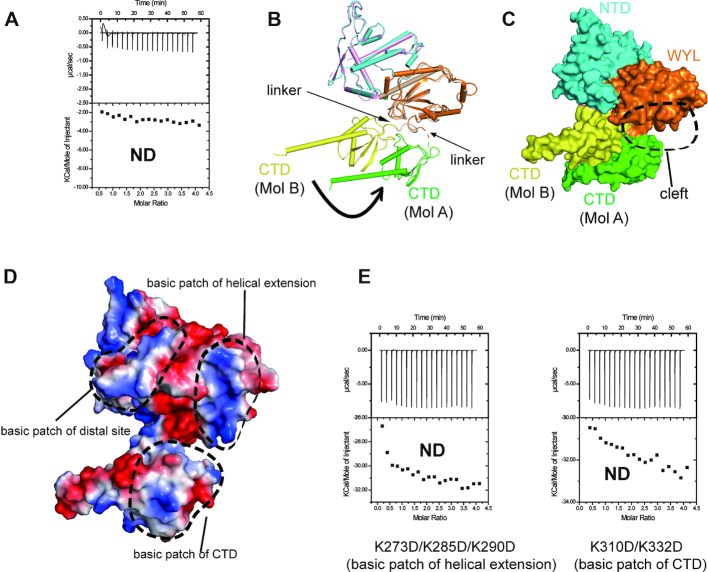
CTD of RspWYL undergoes conformational change. (**A**) ITC studies showed that deletion of CTD abolishes the ssRNA binding. (**B**) Structural comparison of the two molecules of the RspWYL1 homodimer. Significant conformational changes of CTD are observed. (**C**) Surface presentation of the two RspWYL1 molecules depicting the CTD movements. (**D**) Electrostatic surface of Mol B of RspWYL1, with potential RNA binding surfaces highlighted by dotted circles. (**E**) ITC analysis showing that combinational point mutations of the expanded positively charged surface impair ssRNA binding. ND: no detectable binding.

The CTD of RspWYL1 consists of a three-stranded β-sheet surrounded by helices (Figure 1E). Sequence analysis indicated that the CTD shows no sequence homology to proteins with known structures. However, the Dali search revealed an unexpected structural similarity with the activation peptide of Thrombin-activatable fibrinolysis inhibitor (TAFI) known to attenuate fibrinolysis (33). The activation peptide and the CTD of RspWYL1 closely resemble each other, though their C-terminal α-helices point to almost reverse directions in their overall structures (Supplementary Figure S4A and B). A ΔCTD deletion mutant did not bind ssRNA (Figure 5A), suggesting the importance of the CTD in the modulation of RspWYL1’s activity.

Within the RspWYL1 homodimer, the relative orientations of the two CTDs differ significantly (Figure 5B). A stable linker connecting the WYL domain and the CTD in one RspWYL1 protomer (denoted Mol B) can be clearly traced in our electron density map. However, the linker in the other RspWYL1 protomer (denoted Mol A) is flexible with poor electron density, resulting in a rotational movement of the CTD relative to the WYL domain in Mol A (Figure 5B). Remarkably, the conformational change creates a cleft between the WYL domain and the CTD domain of Mol A (Figure 5C), thereby bridging the positively charged patches of these two domains (Figure 5D). This expanded positively charged surface would at least partially contribute to the robust and specific binding capability of RspWYL1 toward ssRNA, thereby boosting the overall efficiency of the Type VI-D CRISPR-Cas system. Consistently, the triple mutation K273D/K285D/K290D in the WYL domain or the double mutation K310D/K332D in the CTD domain abolished the ssRNA binding ability of RspWYL1 (Figure 5E), indicating the crucial role of the expanded positively charged surface formed by the WYL and CTD domains in RNA binding by RspWYL1 in the CRISPR-Cas13d system. Thus, these mutants that impair ssRNA binding may fail to stimulate Cas13d-mediated RNA cleavage by RspWYL1. Regulation of the RNA binding activity of RspWYL1 by the CTD may be analogous to the activation of TAFI, in which the release of the activation peptide of TAFI can activate TAFI (14). This structural insight suggests future directions in further study of the regulation of the RspWYL1 activity.

### Interactions between RspWYL1 and Cas13d

RspWYL1 can enhance the cleavage activities of RspCas13d and EsCas13d, though these two orthologs share a low amino acid sequence similarity (∼38% identities) (9). Under our ITC conditions, RspWYL1 interacts weakly with RspCas13d or EsCas13d (Supplementary Figure S5). Apo Cas13d has a flexible conformation, whereas the binding of crRNA would stabilize Cas13d (13). Likewise, the presence of crRNA could potentially strengthen the interaction between RspWYL1 and Cas13d. It is likely that RspWYL1 may bridge the interaction between Cas13d and ssRNA for both the cis- and trans-cleavage activities. Alternatively, RspWYL1 may increase the local concentration of RNA to facilitate the access of RNA substrates to Cas13d. Consistent with this, RspWYL1 can stimulate both the cis- and trans-cleavage activity of Cas13d in a dose-dependent manner (9). Additionally, the presence of other WYL domain-containing proteins in CRISPR-Cas systems (16,39) implies that the RNA-binding function of the WYL domain plays a general role in modulating various CRISPR-Cas systems.

In brief, the structural insights and biophysical analysis presented in this study of RspWYL1 will serve as a structural framework to elucidate the biological significances and mechanistic roles of the WYL-domain containing accessory proteins in various CRISPR-Cas systems, and thereby advance our understanding of the CRISPR-Cas genome editing strategies.

## DATA AVAILABILITY

Coordinates and structure factor for RspWYL1 have been deposited into the Protein Data Bank under the accession code 6OAW.

## Supplementary Material

gkz269_Supplemental_File
