# Supplementary material for: Structural insights into the modulatory role of the accessory protein WYL1 in the Type VI-D CRISPR-Cas system
Source: Nucleic Acids Res. 2019 Apr 12;47(10):5420–8. doi: 10.1093/nar/gkz269 (PMC6547453; doi:10.1093/nar/gkz269)
Supplement: gkz269_Supplemental_File [file gkz269_supplemental_file.pdf]

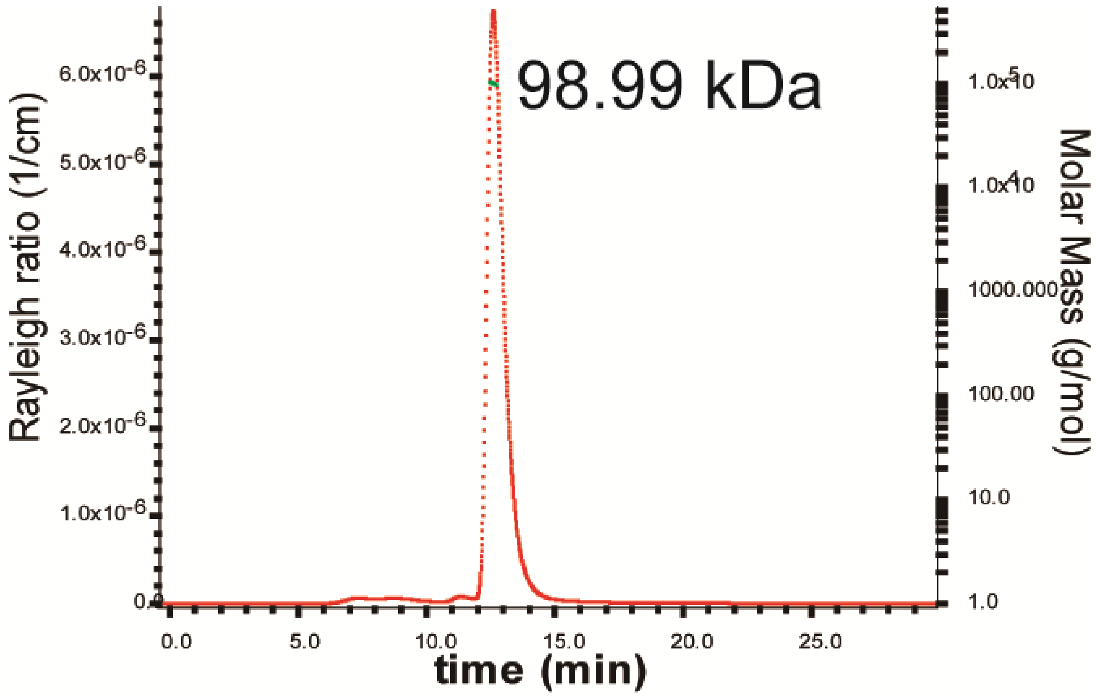

Fig S1. RspWYL1 behaves as dimer in solution. The theoretical molecular weight of monomeric RspWYL1 is 45 kDa; The HPLC SEC-MALS analysis on purified RspWYL1 directly demonstrates that RspWYL1 proteins exist as a homogeneous population of homodimers in solution. The hydrodynamic radius of RspWYL1 in solution is 3.899 nm (+/-0.469%).

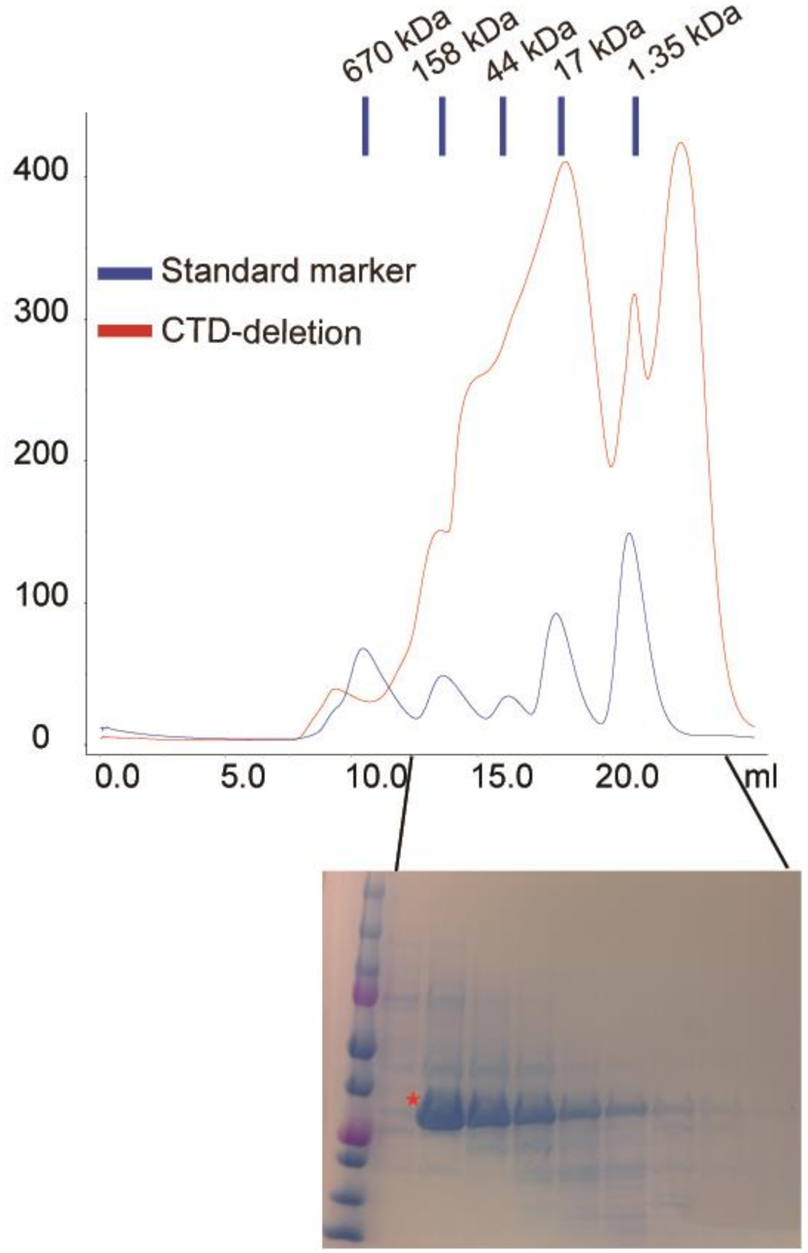

Fig S2. CTD deletion proteins are not monodisperse in gel-filtration chromatography. (top panel) Overlaid size-exclusion chromatograms (SEC) of ΔCTD RspWYL1 (amino acids 3–301) and protein molecular mass standard. (bottom panel) SDS/PAGE analysis of the SEC elution fractions corresponding to ΔCTD. The CTD deletion proteins eluted with a long profile in the gel-filtration, suggesting unstable of the proteins.

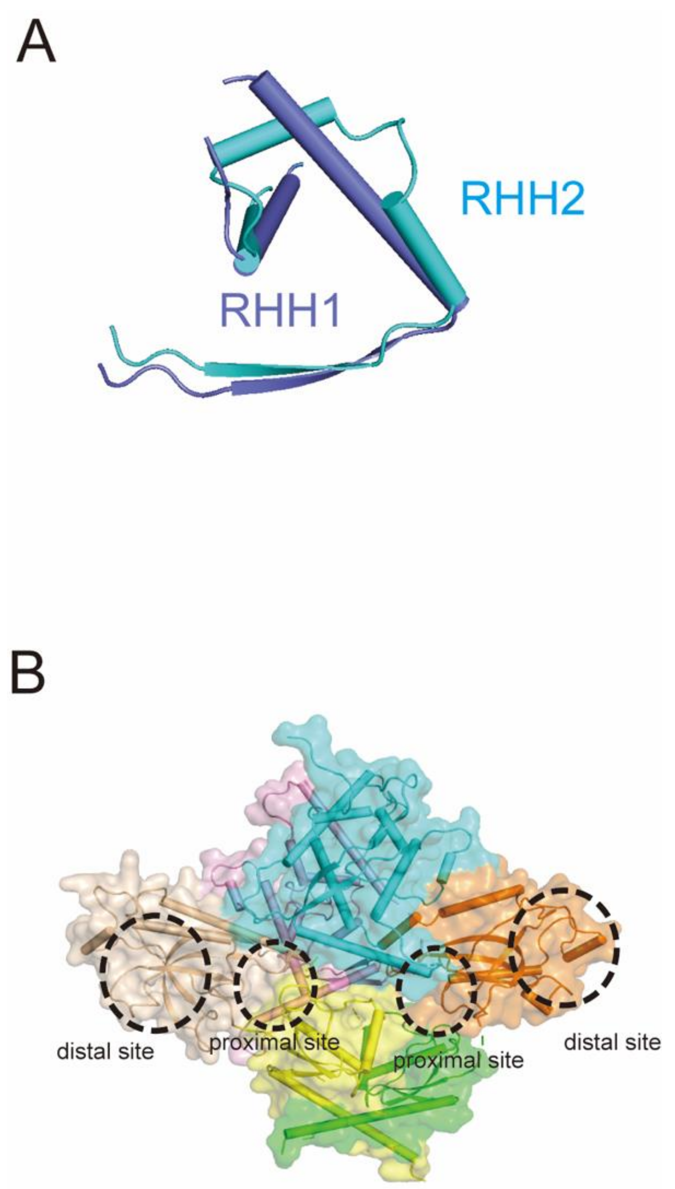

Fig S3. Structural features of NTC and WYL domains.

(A) Structural alignments of the two RHH motifs in RspWYL1.

(B) The proximal and distal sites equivalent to Hfq in RspWYL1 are indicated in circles.

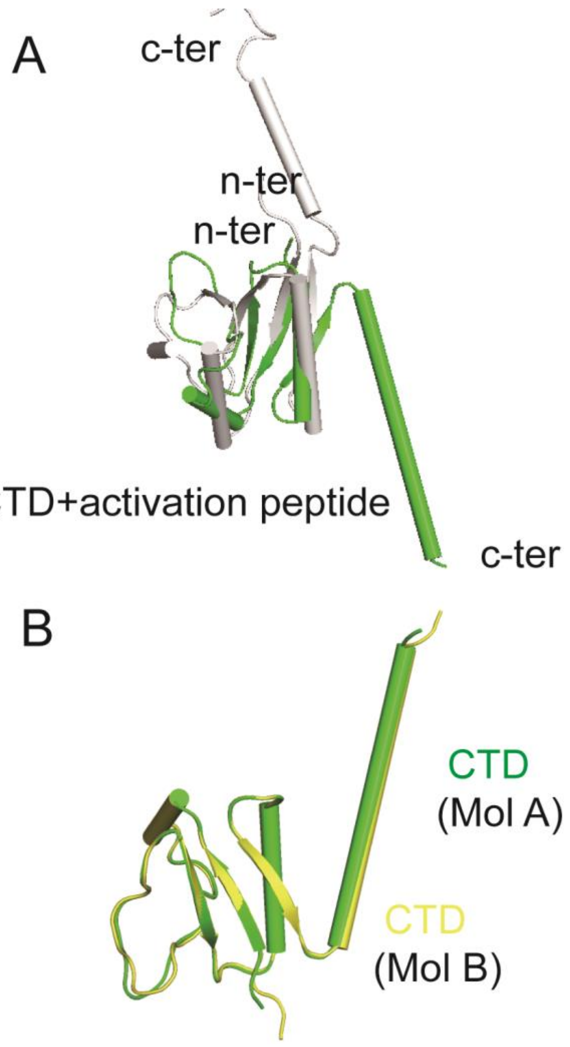

Fig S4. Structure features of CTD

(A) Structure superposition of CTD (green) and activation peptide of Thrombin-activatable fibrinolysis (grey, PDB: 5HVG).

(B) Comparison of the CTDs in the two chains.

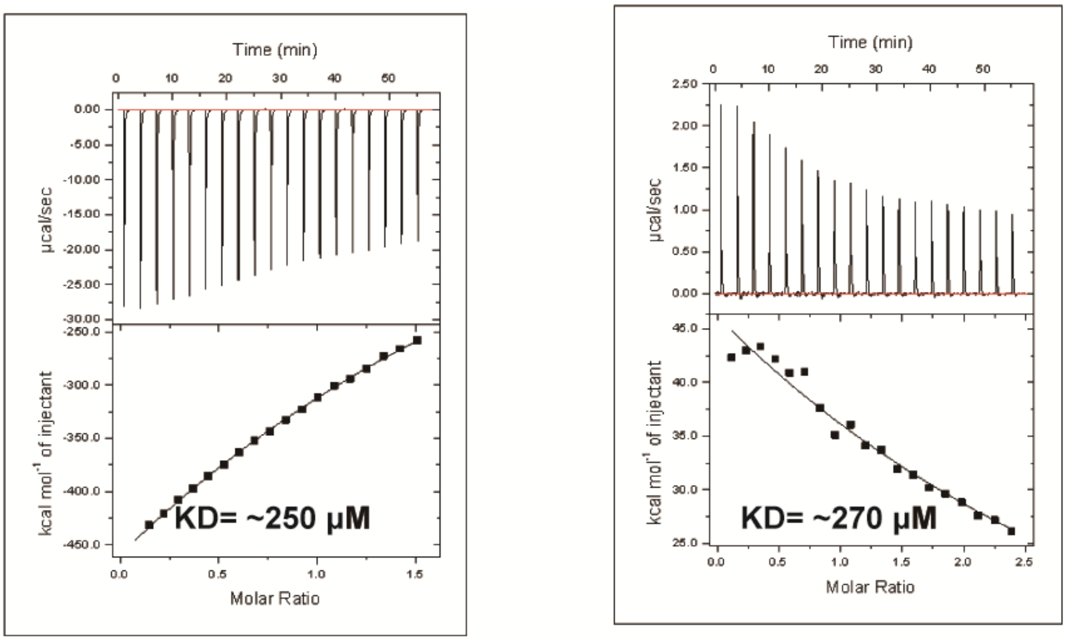

RspWYL1 Titrated into RspCas13d   RspWYL1 Titrated into EsCas13d

Fig S5. Dissociation constants of RspWYL1 with RspCas13d or EsCas13d. ITC-based measurements quantifying the binding affinities between the RspWYL1 and RspCas13d or EsCas13d.

Table S1. Thermodynamics of intermolecular binding events of wild-type RspWYL1 or mutants with various nucleic acids or Cas13d orthologues.

**Intermolecular binding thermodynamics**	**Enthalpy change (ΔH)**	**Entropy change (ΔS)**	**Exothermic or Endothermic**
**dsDNA titrated into RspWYL1**	$\Delta H = 5.277 \times 10^4$ cal/mol	$\Delta S = 191$ cal/mol/deg	Endothermic
**ssDNA titrated into RspWYL1**	$\Delta H = 3.636 \times 10^6$ cal/mol	$\Delta S = 1.22 \times 10^4$ cal/mol/deg	Endothermic
**ssRNA titrated into RspWYL1**	$\Delta H = 1.30 \times 10^4$ cal/mol	$\Delta S = 65.1$ cal/mol/deg	Endothermic
**ssRNA titrated into *RspWYL1$^{R131D/R133D}$***	ND	ND	ND
**ssRNA titrated into *RspWYL1$^{R219D/R221D}$***	$\Delta H = 3.67 \times 10^8$ cal/mol	$\Delta S = 1.23 \times 10^6$ cal/mol/deg	Endothermic
**ssRNA titrated into *RspWYL1$^{\Delta CTD}$***	ND	ND	ND
**ssRNA titrated into *RspWYL1$^{K273D/K285D/K290D}$***	ND	ND	ND
**ssRNA titrated into *RspWYL1$^{K310D/K332D}$***	ND	ND	ND
**RspWYL1 titrated into RspCas13d**	$\Delta H = -1.70 \times 10^6$ cal/mol	$\Delta S = -5.69 \times 10^3$ cal/mol/deg	Exothermic
**RspWYL1 titrated into EsCas13d**	$\Delta H = 4.05 \times 10^5$ cal/mol	$\Delta S = 1.38 \times 10^3$ cal/mol/deg	Endothermic

* ND denotes no significant undetectable binding activity by ITC. 0.5-1mM samples in springe were injected into 0.05-0.1mM samples in cell chamber of the microcalorimeter at 25 °C.
